# Supplementary material for: Lineage tracing of genome-edited alleles reveals high fidelity axolotl limb regeneration
Source: eLife. 2017 Sep 16;6:e25726. doi: 10.7554/eLife.25726 (PMC5621835; doi:10.7554/eLife.25726)
Supplement: Supplementary file 1. [file elife-25726-supp1.docx]

**Supplementary Table 1**

**EGFP**

Target:

GGGCACGGGCAGCTTGCCGG

Universal R

GTGGTGCCCATCCTGGTCGAGCTGG

| Limb Specific Primers **Animal** | Primary Limb | Secondary | Tertiary |
| --- | --- | --- | --- |
| 1 | AATTGTAGGTCAGGGTGGTCACGA | TTCCTTGTAGGTCAGGGTGGTCACGA | GGTTTTGTAGGTCAGGGTGGTCACGA |
| 2 | AAGGGTAGGTCAGGGTGGTCACGA | TTGGTTGTAGGTCAGGGTGGTCACGA | GGAATTGTAGGTCAGGGTGGTCACGA |
| 3 | AACCGTAGGTCAGGGTGGTCACGA | CCAATTGTAGGTCAGGGTGGTCACGA | CCGGTTGTAGGTCAGGGTGGTCACGA |
| 4 | TTAATTGTAGGTCAGGGTGGTCACGA | CCTTTTGTAGGTCAGGGTGGTCACGA | GGCCTTGTAGGTCAGGGTGGTCACGA |

Non-mutant control

AGAGTTGTAGGTCAGGGTGGTCACGA

Negative control

TATATTGTAGGTCAGGGTGGTCACGA

**Meth T1**

Target

GAACGGTCCTTATAGAACAG

Universal F AGCCTCCTTAAAGACAGCCGATATGCT

| **Animal** | **Primary Limb** | **Secondary** | **Tertiary** |
| --- | --- | --- | --- |
| 1 | AAGGTCAACCTGGAGAAGTTCCTGAAGGA | GGTTTCAACCTGGAGAAGTTCCTGAAGGA | CCAATCAACCTGGAGAAGTTCCTGAAGGA |
| 2 | AACCTCAACCTGGAGAAGTTCCTGAAGGA | TTGGTCAACCTGGAGAAGTTCCTGAAGGA | GGCCTCAACCTGGAGAAGTTCCTGAAGGA |
| 3 | AAGGCGATATGCTCTCTGAACGGTCCTTAT | TTGGCGATATGCTCTCTGAACGGTCCTTAT | GGCCCGATATGCTCTCTGAACGGTCCTTAT |

Non-mutant

CCAACGATATGCTCTCTGAACGGTCCTTAT

No DNA

CCGGCGATATGCTCTCTGAACGGTCCTTAT

**Meth T2**

Target

GGAGAAGTTCCTGAAGGAGG

Universal R CACCTCAACGACTGCCGTCCACT

| **Animal** | **Primary Limb** | **Secondary** | **Tertiary** |
| --- | --- | --- | --- |
| 1 | AATTCGATATGCTCTCTGAACGGTCCTTAT | TTCCCGATATGCTCTCTGAACGGTCCTTAT | GGAACGATATGCTCTCTGAACGGTCCTTAT |
| 2 | AACCCGATATGCTCTCTGAACGGTCCTTAT | TTAACGATATGCTCTCTGAACGGTCCTTAT | GGTTCGATATGCTCTCTGAACGGTCCTTAT |
| 3 | AAGGCGATATGCTCTCTGAACGGTCCTTAT | TTGGCGATATGCTCTCTGAACGGTCCTTAT | GGCCCGATATGCTCTCTGAACGGTCCTTAT |

Non-mutant

CCAACGATATGCTCTCTGAACGGTCCTTAT

No DNA

CCGGCGATATGCTCTCTGAACGGTCCTTAT

**Mcherry T1**

Target

GGCGGTCTGGGTGCCCTCGT

Universal F GGACAGGATGTCCCAGGCGAAG

| **Animal** | **Primary Limb** | **Secondary Limb** |
| --- | --- | --- |
| 1 | AATTCGCTTCAAGGTGCACATGGAGG | TTCCCGCTTCAAGGTGCACATGGAGG |
| 2 | AACCCGCTTCAAGGTGCACATGGAGG | TTAACGCTTCAAGGTGCACATGGAGG |
| 3 | AAGGCGCTTCAAGGTGCACATGGAGG | TTGGCGCTTCAAGGTGCACATGGAGG |

Non-mutant control

GGAACGCTTCAAGGTGCACATGGAGG

No DNA Control

GGTTCGCTTCAAGGTGCACATGGAGG

**Mcherry T2**

GTGATGAACTTCGAGGACGG

Universal GTGCCGCGCAGCTTCACCTT

| **Animal** | **Primary Limb** | **Secondary Limb** |
| --- | --- | --- |
| 1 | AACCCGACATCCCCGACTACTTGAAGCTG | TTGGCGACATCCCCGACTACTTGAAGCTG |
| 2 | AAGGCGACATCCCCGACTACTTGAAGCTG | GGAACGACATCCCCGACTACTTGAAGCTG |
| 3 | TTCCCGACATCCCCGACTACTTGAAGCTG | GGTTCGACATCCCCGACTACTTGAAGCTG |

Non-mutant control

AATTCGACATCCCCGACTACTTGAAGCTG

No DNA Control

GGCCCGACATCCCCGACTACTTGAAGCTG

**Tyrosinase**

Target

GGACTTCACTATCCCCTACT

Universal F CGGGAGATCCAGAAGGTGAC

| **Animal** | **Primary Limb** | **Secondary Limb** | **Tertiary Limb** |
| --- | --- | --- | --- |
| 1 | AATTGGCTGAGTAAGTTGGCCAC | TTAAGGCTGAGTAAGTTGGCCAC | GGCCGGCTGAGTAAGTTGGCCAC |
| 2 | AACCGGCTGAGTAAGTTGGCCAC | TTGGGGCTGAGTAAGTTGGCCAC | CCAAGGCTGAGTAAGTTGGCCAC |
| 3 | AAGGGGCTGAGTAAGTTGGCCAC | GGAAGGCTGAGTAAGTTGGCCAC | CCTTGGCTGAGTAAGTTGGCCAC |
| 4 | GGTTGGCTGAGTAAGTTGGCCAC | CCGGGGCTGAGTAAGTTGGCCAC |  |
